# Anti-Epileptic Drug Target Perturbation and Intracranial Aneurysm Risk: Mendelian Randomization and Colocalization Study

**DOI:** 10.1161/STROKEAHA.122.040598

**Published:** 2022-10-27

**Authors:** Mark K. Bakker, Tijmen van Straten, Michael Chong, Guillaume Paré, Dipender Gill, Ynte M. Ruigrok

**Affiliations:** 1 Department of Neurology and Neurosurgery, University Medical Center Utrecht Brain Center, Utrecht University, the Netherlands (M.K.B., T.v.S., Y.M.R.).; 2 Population Health Research Institute; Thrombosis and Atherosclerosis Research Institute; Department of Pathology and Molecular Medicine, McMaster University, Hamilton, Ontario (M.C., G.P.).; 3 Department of Epidemiology and Biostatistics, School of Public Health, Imperial College London, United Kingdom (D.G.).

**Keywords:** aneurysm, genetics, hypertension, risk factors, stroke

## Abstract

In a genome-wide association study of intracranial aneurysms (IA), enrichment was found between genes associated with IA and genes encoding targets of effective anti-epileptic drugs. Our aim was to assess if this pleiotropy is driven by shared disease mechanisms that could potentially highlight a treatment strategy for IA.

**Methods:**

Using 2-sample inverse-variance weighted Mendelian randomization and genetic colocalization analyses we assessed: (1) if epilepsy liability in general affects IA risk, and (2) whether changes in gene- and protein-expression levels of anti-epileptic drug targets in blood and arterial tissue may causally affect IA risk.

**Results:**

We found no overall effect of epilepsy liability on IA. Expression of 21 genes and 13 proteins corresponding to anti-epileptic drug targets supported a causal effect (*P*<0.05) on IA risk. Of those genes and proteins, genetic variants affecting *CNNM2* levels showed strong evidence for colocalization with IA risk (posterior probability>70%). Higher *CNNM2* levels in arterial tissue were associated with increased IA risk (odds ratio, 3.02; [95% CI, 2.32–3.94]; *P*=3.39×10^−^^16^). CNNM2 expression was best proxied by rs11191580. The magnitude of the effect of this variant was greater than would be expected if systemic blood pressure was the sole IA-causing mechanism in this locus.

**Conclusions:**

*CNNM2* is a driver of the pleiotropy between IA and anti-epileptic drug targets. Administration of the anti-epileptic drugs phenytoin, valproic acid, or carbamazepine may be expected to decrease *CNNM2* levels and therefore subsequently decrease IA risk. *CNNM2* is therefore an important target to investigate further for its role in the pathogenesis of IA.

Intracranial aneurysms (IA) are localized dilations of cerebral blood vessels. They are common with a prevalence of 3.2% in the general population.^1^ Rupture of IA leads to aneurysmal subarachnoid hemorrhage, a severe type of stroke with devastating effects; one third of patients die and one third remain dependent on help for daily-life activities.^2^ Of the one third of patients who regain independence and return home, 95% are not able to resume all premorbid activities due to persisting cognitive symptoms.^3,4^ Subarachnoid hemorrhage can be prevented by invasive endovascular or surgical treatment of aneurysms, while therapeutic drug targets are currently lacking.^5^

Both genetic and clinical risk factors, especially smoking and hypertension, contribute to the disease.^6^ Genome-wide association studies (GWAS) identified multiple common genetic variants associated with IA risk.^7–10^ In the largest GWAS to date, including 10 754 cases and 306 882 controls, drug target enrichment showed pleiotropy between IA and anti-epileptic drugs.^9^ We cannot yet explain what drives this pleiotropy. Understanding the mechanisms involved in this pleiotropy will lead to further understanding of the disease-causing mechanisms of IA and may help to identify methods of IA prevention using anti-epileptics or related drugs.

Therefore, the aim of our study was to gain insight in the previously established pleiotropy between IA and anti-epileptic drugs. We hypothesized that there are 2 potential explanations for the observed pleiotropy: there may be a causal effect of genetic predisposition to epilepsy on IA and/or there may be a causal effect of expression levels of genes encoding effective anti-epileptic drugs on IA risk. These hypotheses can both be tested using 2-sample Mendelian randomization (MR) and colocalization analyses.

## Methods

All data used for this study is publicly available and data generated is provided in the article and its supplemental files. Only aggregated data was used and all participants of the original studies provided written informed consent.

### Study Design

As primary analyses we used MR to test the following: (1) the effect of epilepsy liability on IA risk, and (2) the effects of gene- and protein-expression levels of (genes encoding) anti-epileptic drug targets on IA. Upon identifying MR evidence of a causal effect, we performed colocalization analysis to confirm that the exposure and outcome were regulated by the same causal variant. We further investigated whether the identified gene and protein expression levels with MR evidence of causal effects found in analysis 2 may be specific to cases with ruptured IA (ie, aneurysmal subarachnoid hemorrhage) or unruptured IA only, or may affect extracranial subtypes abdominal aortic aneurysms and thoracic aortic aneurysms.

### Dataset Description

As outcome for primary analyses 1 and 2, we used summary statistics of the largest GWAS of IA.^9^ From this dataset, we included data from the 7495 cases and 71 934 controls of European ancestry. To study the effect on ruptured and unruptured IA alone, from the same study, we used summary statistics for cases with unruptured (N=2070) or ruptured (N=5425) IA separately, versus controls (N=71 934).^9^ Furthermore, we obtained summary statistics for cases with thoracic aortic aneurysm (N=765 cases and 874 controls) and abdominal aortic aneurysm (N=4972 cases and 99 858 controls) from individuals of European ancestry.^11,12^

As exposure for MR analysis 1, we used GWAS summary statistics for epilepsy,^13^ which include 15 212 cases with generalized or focal epilepsy and 29 677 controls, with 95% of individuals being of European ancestry. Summary statistics of European ancestry only were unfortunately not available. For analysis 2 and subsequent analyses, we obtained expression quantitative trait loci (eQTL) in whole blood and arterial tissue and protein quantitative trait loci (pQTL) in blood serum or blood plasma from 6 studies.^14–21^ No eQTL datasets of intracranial arteries were available for our analyses, so data derived from other (extracranial) arteries, being tibial artery, coronary artery, and aorta, were used instead. Study names and sample size are given in the Supplemental Detailed Methods. Where possible, we selected eQTL and pQTL datasets including European ancestry individuals only. However, this was not possible for data from the ORIGIN trial (Outcome Reduction With Initial Glargine Intervention; with 47% of participants being of European ancestry and the remaining being admixed Latino with a predominantly European ancestral component) and GTEx version 8 (with 85% of participants being European ancestry).^22^ There was no known sample overlap between studies jointly analyzed with MR.

We further used summary statistics for diastolic and systolic blood pressure from the UK Biobank provided by the Neale lab (http://www.nealelab.is/uk-biobank/). Summary statistics from inverse-rank normalized blood pressure levels were used.

### Selecting Anti-Epileptic Drug Targets

Our previous GWAS identified pleiotropy between the anti-epileptic drug class, which consist of 38 anti-epileptic drugs, and IA.^9^ We selected gene targets for these 38 anti-epileptic drugs using the drug-gene interaction database and the connectivity map (CMap) resource (Table S1).^23,24^ Details of the selection procedure are given in the Supplemental Detailed Methods.

### Mendelian Randomization

The inverse variance weighted (IVW) MR method was selected as our main MR analysis method. Mendelian Randomization Pleiotropy Residual Sum and Outlier (MR-PRESSO) was used in addition to exclude eQTL and pQTL with potential pleiotropic effect. The assumptions regarding the interpretation of MR and how we accounted for those as well as the variant selection are outlined in the Supplemental Detailed methods.

Drug targets with a *P*-value <0.05 for the IVW method, and that did not have a *P*-value >0.05 for MR-PRESSO (since this analysis requires 2 additional independent variants, which were not available for all QTL, absence of an MR-PRESSO estimate was no disqualifying threshold) were selected for colocalization analysis.

### Sensitivity Analyses

Several sensitivity analyses were performed to determine the presence of heterogeneity and pleiotropy for genetic variants or studies. We performed the following analyses: MR-Egger, weighted median, weighted mode, simple mode, Cochran’s Q test, and the Steiger directionality test. More details and rationale can be found in the Supplemental Detailed Methods.

### Colocalization Analysis

To confirm that eQTL or pQTL with a putative causal effect on IA as identified by MR (defined as having a p-value for IVW and if available MR-PRESSO<0.05) share a causal genetic variant, we performed colocalization analysis using R package Coloc (version 3.2-1).^25^ The variant most strongly associated with the exposure in the MR analysis (ie, with the lowest *P*-value) was selected as reference variant. Variants±100 kb of the reference variant were included. The 1000 Genomes v3 European ancestry dataset was used as LD reference panel. Evidence for colocalization was defined as a posterior probability for a shared causal variant greater than 0.7 (posterior probability of hypothesis 4>0.7).

### Inferring the Direction of Effect of Anti-Epileptic Drugs on IA Risk

For drugs targeting expression of the genes and proteins with MR evidence of an effect on IA risk, we assessed the direction of effect. From the CMap database, we obtained similarity scores between (1) gene expression after administration of a drug, and (2) either overexpression or knockdown of a gene. Similarity scores can be positive (similar expression patterns) or negative (opposite expression patterns). If knockdown of a gene results in highly similar expression as administration of a drug and higher expression of that gene increases risk of IA, one can deduce that administration of the drug is expected to reduce risk of IA. Using information from CMap and the direction of MR effect, we determined the expected direction of IA risk after administration of anti-epileptic drugs.

### Assessing and Benchmarking the Role of Blood Pressure in the *CNNM2* Locus

We performed additional MR analyses to (1) determine the role of blood pressure as potential causative mechanism for IA in the *CNNM2* locus, and to (2) compare the importance of genetically proxied *CNNM2* expression for IA risk against all other genes in the genome. These analyses were performed using MR as described above and are explained further in the Supplemental Detailed Methods.

## Results

### Effect of Epilepsy Risk on IA Risk

No MR evidence of a causal effect of epilepsy liability on IA risk was found (odds ratio [OR], 1.30; [95% CI, 0.63–2.67]; *P*=0.474; Table S2).

### Effect of Anti-Epileptic Drug Targets on IA Risk

In drug-gene interaction database, 37 out of our 38 candidate anti-epileptic drugs were listed, while in CMap only 15 of these 38 drugs were listed. Both databases did not include valpromide. In total, 537 anti-epileptic drug targets were identified using drug-gene interaction database and CMap (Table S1). After selecting eQTL and pQTL with at least 2 statistically significant LD-independent variants in a tissue to allow the IVW method, 534 eQTL or pQTL for 414 unique targets remained for MR at the *P*<5×10^−^^5^ level, and 270 eQTL or pQTL for 250 unique targets at the *P*<5×10^−^^8^ level (Tables S3–S5). For 423 eQTL or pQTL, only a Wald ratio test could be performed at the *P*<5×10^−^^5^ level, and 365 at the *P*<5×10^−^^8^ level.

**Figure 1.**
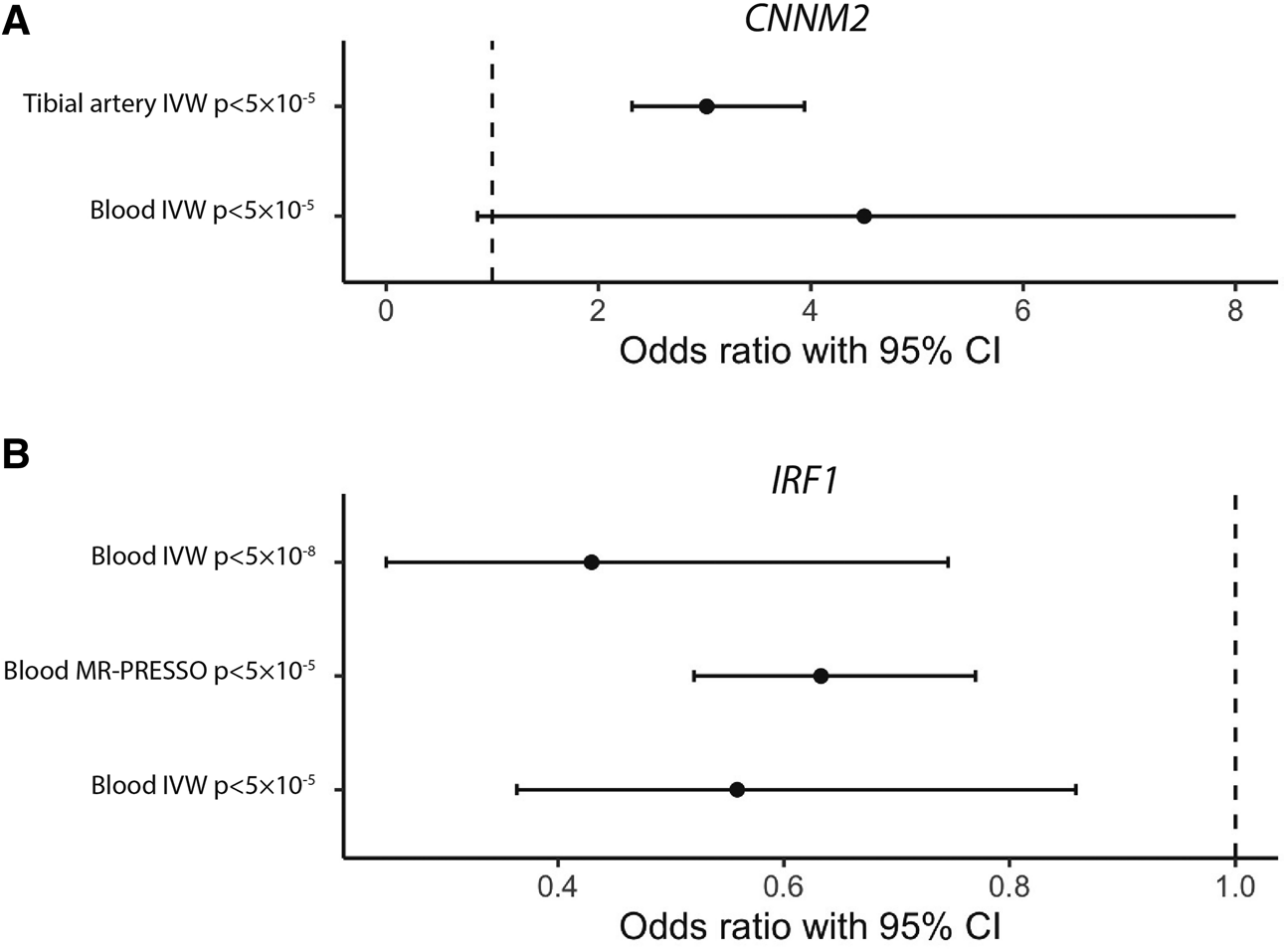
**Mendelian randomization analysis results for *CNNM2* and *IRF1* gene expression on intracranial aneurysm risk.** *CNNM2* indicates cyclin and CBS domain divalent metal cation transport mediator 2; *IRF1*, interferon regulatory factor 1; IVW, inverse variance weighted method; and MR-PRESSO, Mendelian Randomization Pleiotropy Residuals Sum and Outlier.

A total of 21 eQTL and 13 pQTL showed at least a suggestive statistically significant effect (*P*<0.05) according to the IVW method (Tables S3–S5). Out of these 34 drug targets, 2 eQTL also showed evidence for a shared causal variant with IA (Figure S1): higher *CNNM2* levels in tibial artery were associated with a risk-increasing effect with statistical significance after correcting for the number of genes and proteins tested (at the *P*<5×10^−^^5^ level: OR, 3.02 per number of transcripts-per-million increase; [95% CI, 2.32–3.94]; *P*=3.39×10^−^^16^; posterior probability of a shared causal variant=0.94) and higher *IRF1* levels in blood were associated with a protective effect at nominal significance (at the *P*<5×10^−^^5^ level: OR, 0.63 per unit increase of trimmed-mean of M value normalized expression; [95% CI, 0.52–0.77]; *P*=0.0038; at the *P*<5×10^−^^8^ level: OR, 0.43; [95% CI, 0.25–0.75]; *P*=0.0027; posterior probability of a shared causal variant=0.79; Tables 1 and 2, Figure 1). For the other arterial tissues of the aorta and coronary artery, no eQTL for *CNNM2* were available while for the *P*-value threshold of 5×10^−^^8^ no eQTL for tibial artery remained. In blood, the effect of *CNNM2* on IA risk was larger than in tibial artery tissue but statistically nonsignificant (at the *P*<5×10^−^^5^ level: OR, 4.50 per transcripts-per-million; [95% CI, 0.86–23.6]; *P*=0.075). The effect of *IRF1* could not be assessed in arterial tissue because insufficient genetic variants were available.

**Table 1.**
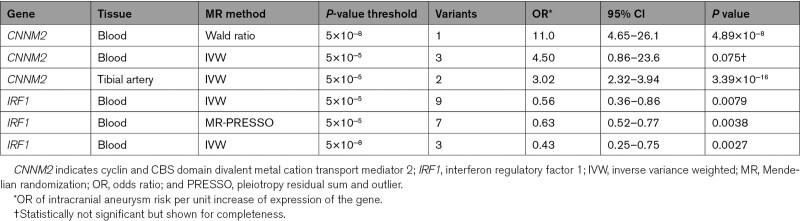
Mendelian Randomization Results of Gene-Tissue Pairs With a Statistically Significant Effect on Intracranial Aneurysm Risk, and Evidence for a Shared Causal Variant With Intracranial Aneurysms

**Table 2.**
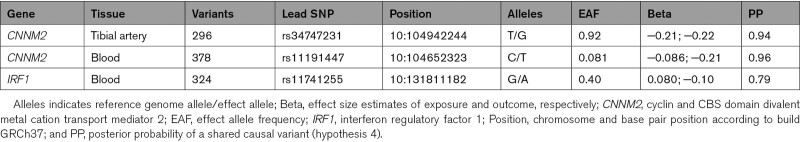
Colocalization Analysis Results Between Gene Expression and Intracranial Aneurysm Risk of Genes With Posterior Probability for a Shared Causal Variant >0.7 Between Its Expression and Intracranial Aneurysm Risk

Results of sensitivity analyses are shown in Tables S3–S5. The Cochran’s Q test indicated there was no evidence for heterogeneity in the MR analyses of *CNNM2* and *IRF1* expression levels on IA risk (Table S6). The Steiger directionality confirmed that the genetic associations were consistent with a causal effect of *IRF1* and *CNNM2* levels on IA risk, and not in the opposite direction (Table S7).

Since no statistically significant MR effect was identified using the pQTL data from the ORIGIN trial,^21^ in which a large group of non-European ancestry participants were included, we did not perform additional analyses to rule out bias as a result of population differences between exposure and outcome datasets.

### Effect of *CNNM2* Levels on Other Phenotypes

Next, we tested whether the effect of the *CNNM2* expression level was specific to ruptured or unruptured IA. MR showed a statistically significant effect for *CNNM2* expression on unruptured IA risk in blood (OR, 5.86; [95% CI, 2.17–15.87]; *P*=4.98×10^−^^4^) and tibial artery (OR, 3.11; [95% CI, 1.92–5.06]; *P*=4.52×10^−^^6^; Table 3), both at the *P*<5×10^−^^5^ level. These effects are consistent with the analysis of the combined IA set. For ruptured IA only, a statistically nonsignificant effect of similar magnitude was found for *CNNM2* in blood (OR, 4.01; [95% CI, 0.58–27.6]; *P*=0.159), whereas the effect in arterial tissue could not be established due to lack of genetic variants.

**Table 3.**
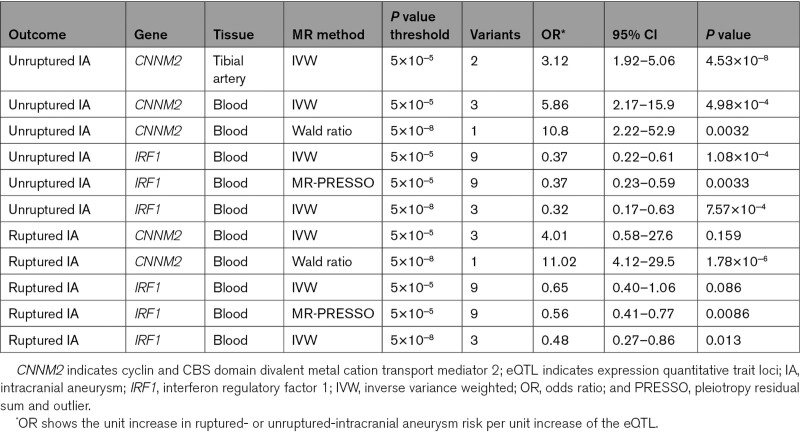
Results of the Mendelian Randomization Analyses With eQTL as Exposure and Ruptured or Unruptured Intracranial Aneurysm as Outcome

No statistically significant effect of *CNNM2* expression level on the risk of aneurysm types abdominal aortic aneurysm and thoracic aortic aneurysm in either blood or arterial tissue was found (Table S8).

### Direction of Effect on IA of Administering Anti-Epileptic Drugs

In Table 4, we report the expected effect direction of administering an anti-epileptic drug on IA risk given the MR effect size and direction, and the CMap direction of effect (overexpression or knockdown). Administration of anti-epileptic drugs phenytoin, valproic acid, or carbamazepine is expected to lead to a decrease in *CNNM2* levels and a subsequent decrease in IA risk.

**Table 4.**
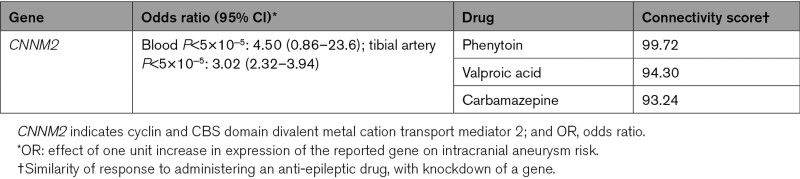
Anti-Epileptic Drugs Targeting Genes With a Suspected Causal Effect on Intracranial Aneurysm Risk

### Assessing the Role of Blood Pressure in the *CNNM2* Locus

The gene *CNNM2* has previously been associated with hypertension, an important risk factor for IA.^26–29^ Colocalization analysis confirmed that a shared causal variant between increased IA risk and increased blood pressure, most likely being SNP rs11191580, in this *CNNM2* locus is responsible for increased *CNNM2* expression levels in tibial artery and in blood (Table S9).

**Figure 2.**
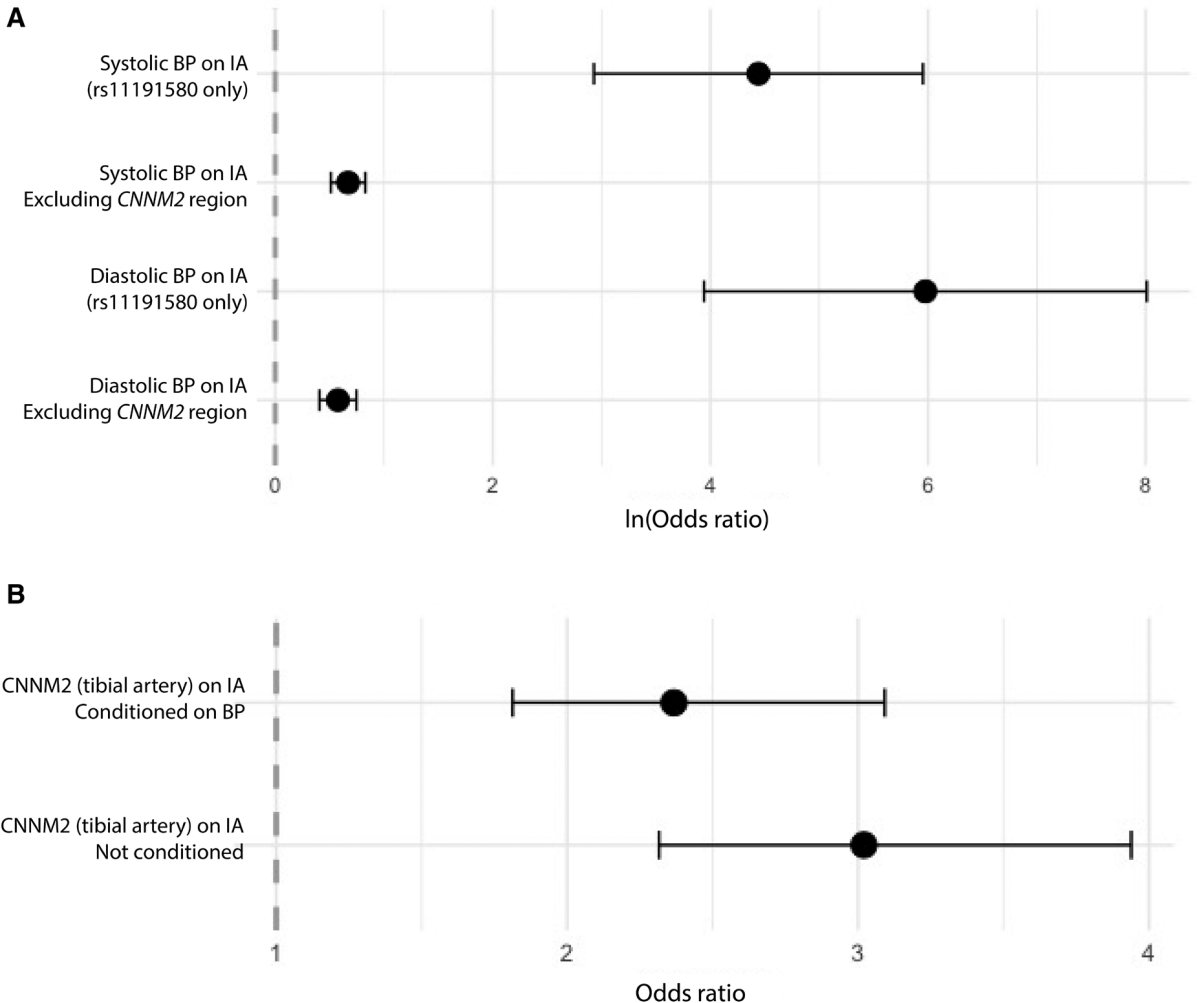
**Mendelian randomization analyses to test the dependance of the CNNM2 locus on blood pressure. A**, Mendelian randomization analysis of genetically proxied systolic blood pressure (SBP) and diastolic blood pressure (DBP) on intracranial aneurysm (IA) risk. SBP and DBP were genetically proxied by only the most likely causal variant in the *CNNM2* locus rs11191580 (first and third row), or genetically proxied by genome-wide variants, excluding the *CNNM2* locus. **B**, Mendelian randomization analysis of genetically proxied *CNNM2* levels in tibial artery on IA risk with and without conditioning on SBP and DBP. Error bars represent 95% CIs. BP indicates blood pressure; and *CNNM2*, cyclin and CBS domain divalent metal cation transport mediator 2.

Next, we tested whether the observed effect of increased *CNNM2* expression in tibial artery on IA risk could be mediated fully through increased blood pressure (ie, increased *CNNM2* expression causing increased systemic blood pressure, which in turn increases IA risk). To this end, we tested whether the MR effect of increased blood pressure on IA proxied by the most likely causal SNP in this *CNNM2* locus, rs11191580, is similar to that proxied by the rest of the variants in the genome. The MR effect of increased blood pressure on IA with rs11191580 as genetic instrument was β=5.98 (95% CI, 3.94–8.01) for diastolic and 4.44 (2.92–5.95) for systolic blood pressure. These MR effect sizes for diastolic and systolic blood pressure were expected to be only 0.58 (0.41–0.75) and 0.67 (0.51–0.83), respectively, if increased blood pressure fully mediated the effect of genetically proxied *CNNM2* expression on IA risk, based on the genome-wide effect of increased blood pressure on IA, excluding the *CNNM2* locus (Figure 2A). After conditioning the IA summary statistics on blood pressure, the effect of genetically proxied increased *CNNM2* levels slightly reduced but remained statistically significant with largely overlapping CIs compared to the nonconditioned effect (nonconditioned OR, 3.02 per transcripts-per-million; [95% CI, 2.32–3.94]; *P*=3.39×10^−^^16^; conditioned OR, 2.37; [95% CI, 1.81–3.09]; *P*=2.55×10^−^^10^; Figure 2B).

### Benchmarking the Role of *CNNM2* in Blood Pressure and IA

In our exome-wide analysis, of the genes associated with diastolic or systolic blood pressure, *CNNM2* levels in tibial artery had the greatest effect size on IA risk (OR, 1.07, [95% CI, 1.06–1.09]; Figures S2 and S3; Table S10). In blood, *CNNM2* levels had no statistically significant effect on IA despite much larger sample size (N=584 for tibial artery, versus N=31 684 for blood), and 1 additional LD-independent variant (OR, 1.06, [95% CI, 0.99–1.12]). This indicates that compared to all other genes, differential *CNNM2* expression has a more important role in IA risk, and this role is bigger in arterial tissue than in blood.

## Discussion

We found that the previously established pleiotropy between genetic risk for IA and the genes targeted by anti-epileptic drugs is not driven directly by an increased predisposition for epilepsy also affecting IA risk but is partly explained by variation in gene expression of *CNNM2*. Higher gene expression levels of *CNNM2*, a gene of which knockdown mimics gene expression patterns after anti-epileptic drug administration, in arterial tissue is consistent with a causal increase in the risk of IA. The effect was seen for the combined group of ruptured and unruptured IA, as well as for unruptured and ruptured IA alone, although the effect for ruptured IA was not statistically significant. The anti-epileptic drugs phenytoin, valproic acid, or carbamazepine are expected to lower *CNNM2* levels, which subsequently could result in a decrease of the risk for IA.

The *CNNM2* gene is located within an IA risk locus on chromosome 10q24.32.^9,10^ In a study using chromatin conformation capture in the human circle of Willis, active enhancers within the 10q24.32 locus were identified that link to the promoter of *CNNM2*, prioritizing *CNNM2* as a likely gene involved in the pathogenesis of IA.^30^ In a multitude of genetic association studies across several ancestries, the *CNNM2* locus was also linked to blood pressure and hypertension, a major risk factor for IA.^26–29^ Here, we found that the most likely causal variant for IA (SNP rs11191580) is also associated with increased blood pressure. However, the effect that this variant has on IA risk is orders of magnitude greater than expected when increased systemic blood pressure fully mediates the effect of this variant on IA and the effect of genetically proxied *CNNM2* on IA largely remained after conditioning on blood pressure, suggesting that the *CNNM2* locus contributes through an additional mechanism than increased blood pressure alone to IA risk.

The *CNNM2* gene encodes cyclin and CBS domain divalent metal cation transport mediator 2 responsible for the transport mediation of Mg^2+^, which could be a potential IA-causing mechanism. Variants on the *CNNM2* locus have been linked to serum magnesium ion (Mg^2+^) levels.^31^ Moreover, experiments in zebrafish show that CNNM2 protein function is fundamental for Mg^2+^ homeostasis.^32^ Truncating mutations in CNNM2 have been found to cause low serum Mg^2+^ levels.^33,34^ Lower Mg^2+^ levels are associated with increased risk of hypertension,^35^ a major risk factor for IA, while higher serum Mg^2+^ levels are protective.^36^ However, in mice, loss of *Cnnm2* in the kidney resulted in low serum Mg^2+^ levels and decreased blood pressure.^37^ Therefore, the interplay between Mg^2+^, hypertension, and IA pathogenesis remains to be elucidated.

Here, *CNNM2* was listed as anti-epileptic drug target by expression similarity between *CNNM2* knockdown and administration of anti-epileptic drugs. The gene is not an intended anti-epileptic drug target, but cases have been reported with mutations in *CNNM2* and epileptic seizures, both with and without hypomagnesia.^32,38–40^ Further, cardiovascular effects, in particular low blood pressure, are known side-effects of phenytoin, the drug linked most strongly to *CNNM2*.^41^ These findings support a link between *CNNM2* and off-target effects of anti-epileptic drugs.

Intracranial arteries and blood are the most likely involved tissues in IA development and rupture. We investigated eQTL and pQTL in blood and in 3 extracranial arterial tissues as proxies for intracranial arterial tissue for which no eQTL data are available. The observed effects for *CNNM2* were consistent across these tissues. We found that systemic blood pressure is unlikely the causal mechanism for IA in this locus. Instead, CNNM2 may disrupt a process in the intracranial arterial wall or blood with systemic blood pressure difference as a result instead of as a cause. To study this, the arterial or blood cell-type in which CNNM2 influences IA risk must be identified.

This study has certain limitations. First, not all anti-epileptic drugs were present in the CMap database or DGIbd, and the drug valpromide was not listed in either database. Since many drugs had overlapping targets, we expect that most anti-epileptic drug targets were included. Second, expression of most genes is influenced by local genetic interaction (cis-eQTL).^14^ This means that most variants influencing gene expression are in proximity and thus often in LD. Therefore, only a small number of LD-independent variants could be used for MR, leading to relatively few genes with sufficient LD-independent variants for MR and a relatively large imprecision in MR estimates for the remaining genes. Compared to eQTL, variants affecting protein levels (pQTL) are more often distant from the encoding gene.^42^ This resulted in a greater number of SNPs to be included in our pQTL analyses, partially overcoming the limitation of few instruments. Finally, findings by MR infer a potential causal effect of genetic predisposition of an exposure on an outcome but does not provide evidence that changes in exposure by therapeutical intervention will substantially influence the outcome. Therefore, additional research is needed to determine the effect of the usage of anti-epileptic drugs on IA risk.

In conclusion, we investigated the pleiotropy between IA and anti-epileptic drug targets. We found a causal effect of increased gene expression of anti-epileptic drug target *CNNM2* on IA. The effect could not be explained solely by increased blood pressure. Anti-epileptic drugs phenytoin, valproic acid, and carbamazepine are expected to lower *CNNM2* levels, which subsequently may lead to a lower IA risk. Follow-up studies are required to investigate whether persons exposed to these anti-epileptic drugs have indeed a lower risk of unruptured IA and/or aneurysmal subarachnoid haemorrhage and how variation in *CNNM2* expression can lead to IA. In addition, modifiable risk factors can be studied for their potential in lowering *CNNM2* levels. This study highlights *CNNM2* as a relevant drug target for IA.

## Article Information

### Acknowledgments

The authors would like to thank the International Stroke Genetics Consortium (ISGC) intracranial aneurysm working group for providing GWAS summary statistics for intracranial aneurysms.

### Sources of Funding

We acknowledge the support from the Netherlands Cardiovascular Research Initiative: An initiative with support of the Dutch Heart Foundation, CVON2015-08 ERASE. This project has received funding from the European Research Council (ERC) under the European Union’s Horizon 2020 research and innovation program (grant agreement No. 852173). D.G. is supported by the British Heart Foundation Centre of Research Excellence (RE/18/4/34215) at Imperial College London and a National Institute for Health Research Clinical Lectureship at St George’s, University of London (CL-2020-16-001).

### Disclosures

Dr Gill is employed part-time by Novo Nordisk. Dr Paré reports affiliations with Amgen Inc, Bayer, and Sanofi Pasteur Inc. The other authors report no conflicts.

### Supplemental Material

Supplemental Methods

Checklist

Tables S1–S10

Figures S1–S3

References 43–53

## Supplementary Material
