# Supplementary material for: Anti-Epileptic Drug Target Perturbation and Intracranial Aneurysm Risk: Mendelian Randomization and Colocalization Study
Source: Stroke. 2022 Oct 27;54(1):208–16. doi: 10.1161/STROKEAHA.122.040598 (PMC9794136; doi:10.1161/STROKEAHA.122.040598)
Supplement: Supplementary file 1 [file str-54-208-s001.pdf]

# SUPPLEMENTAL MATERIAL

For the manuscript entitled '*Anti-epileptic drug target perturbation and intracranial aneurysm risk: Mendelian randomization and colocalization study*.'

## Detailed Methods

### *R packages*

All Mendelian randomization analyses, as well as the pre-processing steps linkage disequilibrium clumping and data harmonization were performed in R statistical software (RStudio version 1.3.1093) using the R packages TwoSampleMR (version 0.5.5) and MRInstruments (version 0.3.2). [43] Colocalization analyses were performed using the R package Coloc (version 3.2-1). [25]

### *Dataset description*

Blood eQTL summary statistics were obtained from the eQTLGen consortium (N = 31,684), [14] while aorta (N = 387), tibial (N = 584) and coronary (N = 213) artery tissue eQTL summary statistics were obtained from the genome tissue expression project (GTEx) version 8. [15] The eQTLGen summary statistics contained both cis- and trans-eQTL, while GTEx included only cis-eQTL within a 1 Mb window. Blood plasma pQTL summary statistics were obtained from the INTERVAL study (N = 3,301), [16] cardiovascular Risk in Young Finns Study and FINRISK studies (N = 6,323), [17] KORA F4 study (N = 3,080), [18] and the IMPROVE study (N = 3,711). [19] Blood serum pQTL summary statistics were obtained from the Cardiovascular Risk in Young Finns Study and FINRISK studies (N =2,019), [17] AGES Reykjavik study (N = 5,457), [20] and the ORIGIN trial (N = 4,147). [21] All pQTL summary statistics included both cis- and trans-pQTL.

### *Selection of anti-epileptic drug targets*

Anti-epileptic drug targets were selected based on two resources: the drug-gene interaction database (DGIdb) and the connectivity map (CMap) resource. [23, 24] DGIdb lists genes as potential targets for a drug based on 42 potentially druggable categories and 49 interaction types, which includes established drug-gene interactions and putative interactions. [23] We included all genes that were listed in DGIdb as having a potential interaction with at least one of the 38 anti-epileptic drugs. CMap is a resource that uses gene expression patterns to investigate similarity in responses to various perturbagens. These perturbagens include administration of a drug, overexpression of a gene, and knockdown of a gene. Similarity in gene expression patterns as response to perturbagens are quantified in a connectivity score ranging from -100% (exactly opposite) to +100% (identical). [24] Genes for which overexpression or knockdown resulted in gene expression patterns with a connectivity score >90% or <-90% compared to gene expression patterns as response to administration of any of the anti-epileptic drugs of interest, were selected. If more than 20 genes passed this threshold, the top 20 most similar and dissimilar genes were selected. Genes matching the abovementioned criteria in either DGIdb or CMap were included for further analysis.

### *Mendelian randomization*

In other to infer causality in MR, a genetic variant causing the outcome through the exposure (and therefore being a valid instrumental variable) must meet core three assumptions: 1) genetic variants have a causal effect on the outcome, 2) the genetic variants affect the outcome only through the exposure, and 3) the genetic variants are not associated with a confounding factor influencing both the exposure and outcome. [44]

The inverse variance weighted (IVW) MR method was selected as our main MR analysis method. Two linkage disequilibrium (LD)-independent variants are needed to perform IVW. IVW estimates are accurate if all genetic variants included conform to the MR assumptions of no heterogeneity. If only a single LD-independent variant was available, we used a Wald ratio test to quantify its MR effect but did not use it for further analysis since with a single genetic variant no test for pleiotropy can be performed, making it impossible to assess reliability of the estimates. MR-PRESSO was used in addition to exclude eQTL and pQTL where pleiotropic outliers influenced the IVW estimate. The MR-PRESSO method is an extension of the IVW method and is robust for horizontal pleiotropy amongst the genetic variants compared to IVW, by identifying and removing pleiotropic outliers from the analysis. [45, 46] First, MR-PRESSO was used to identify pleiotropic outliers, which were then removed, and MR-PRESSO was subsequently used to calculate the MR effect using R package MRPRESSO (version 1.0). Empirical p-value was obtained by 1000 permutations. At least four LD-independent variants are needed to perform MR-PRESSO.

Instruments (i.e., uncorrelated variants) for MR should be associated with the exposure. To achieve this, a threshold for association p-value is typically applied. The downside of a stringent threshold is that it limits the number of variants available thereby potentially decreasing accuracy. This means that no single optimal p-value threshold exists. Therefore, we used two p-value thresholds for the association of a variant with the exposure: $p < 5\times10^{-5}$ and $p < 5\times10^{-8}$. After this selection the genetic variants were clumped to select LD-independent instruments (LD $r^2 > 0.01$, or outside 10 megabase window to include) using the 1000 Genomes European reference panel. The analysis was performed using the R packages TwoSampleMR (version 0.5.5) and MRInstruments (version 0.3.2). [43]

## *Colocalization analysis*

If two traits show a genetic association signal on the same genomic region, colocalization analyses can estimate the probability that these signals are driven by the same causal variant. The colocalization approach assumes that 1) there is a maximum of one causal genetic instrument for either trait, 2) the causal probability of a genetic instrument is independent of the causal probability of other genetic instruments in the analysis, and 3) every causal genetic variant (genotyped or imputed) is included in the colocalization analysis. Following these assumptions, there are five hypotheses for each performed analysis: Hypothesis 0 (H0) states there are no causal genetic instruments present for either trait; (H1) states there is one causal genetic instrument present for the trait 1; (H2) states there is one causal genetic instrument present for trait 2; (H3) states both traits have a causal variant in the region, but these are distinct; (H4) states there is one shared causal genetic instrument present for both traits. [25]

COLOC requires two prior probabilities. The first prior defines the probability tan any variant is causal for any trait. This was set to $1\times10^{-4}$ (i.e. one in 10,000 genome-wide genetic instruments are causal for either trait). The second prior defines the probability that any variant is causal for both traits and was set to $1 \times 10^{-6}$. [25]

## *Sensitivity analyses*

The estimation of a causal effect by Mendelian randomization will be biased if the majority of the genetic instruments are invalid or if any of the genetic variants show pleiotropic effects. Therefore, the MR Egger regression, weighted median-based, weighted mode-based, simple mode-based and Mendelian Randomization Pleiotropy RESidual Sum and Outlier (MR-PRESSO) robust methods were performed in extension of the inverse variance weighted method. The claim of any causal effect of the exposure on intracranial aneurysms will be more credible when the results of the MR analyses are consistent. [46]

The MR Egger regression and MR-PRESSO methods detect pleiotropic effects. MR Egger estimates the causal effect from the weighted regression slope, without forcing the intercept through zero. The results of the MR Egger method will be uniform with the effect size estimate of the IVW if the MR-Egger intercept shows a value of zero. This method requires independent pleiotropic effects of the genetic variants associated with the exposure, which allows for every genetic variant used in the MR analysis to have any form of pleiotropic effect. [47] The weakness of the MR Egger method is that the estimation is specifically affected by genetic variant outliers with large effects, and violation of the InSIDE assumption (instrument strength of direct effect, meaning effect of genetic variants should be independent of direct effects of genetic variants on the outcome). [48]

The weighted and simple mode methods require that most genetic variants contribute to the estimation of the true causal effect instead of other occurring effects. So, both weighted- and mode-based methods are more robust to a small number of pleotropic variants compared to the MR-Egger and IVW MR methods. [49, 50]

The Cochran's Q test was done to quantify the heterogeneity in the effects of genetic variants. If heterogeneity is large, this could indicate that Mendelian randomization assumptions are violated. Cochran's Q-test required the genetic variants to be independent. [51]

The Steiger test of directionality is used to infer the causal direction of exposure and outcome. It is robust to measurement error, even when the effect of exposure on outcome is small. MR Steiger requires an assumed direction and then tests whether this direction is consistent with a causal effect. It indicated a causal relationship (in any direction) by testing the hypothesis that the difference in correlations between genetic variant $g$ with exposure $x$ and $g$ with outcome $y$ is greater than expected under the null hypothesis of identical correlations. The direction is inferred from the MR analysis and Steiger test. [52]

### *Assessing the role of blood pressure in the CNNM2 locus*

We tested whether the effect of increased in *CNNM2* gene expression, genetically proxied by the most likely causal variant rs11191580, on IA, could be explained solely by increased blood pressure caused by rs11191580. If this is true, then the MR effect size estimate of blood pressure genetically proxied by only rs1119580 (the exposure) on IA (the outcome) is similar to the MR effect size estimate of blood pressure genetically proxied by all other genetic variants associated with blood pressure combined (the exposure) on IA (the outcome). Therefore, we performed a Wald ratio test of blood pressure (systolic and diastolic) on IA using only variant rs1119580 and an IVW test of genome-wide variants associated with blood pressure (P-value threshold = $5\times10^{-5}$) on IA excluding variants in the *CNNM2* gene +/- 1 megabases.

To further validate that the effect that genetically proxied differential *CNNM2* levels have on IA is not solely mediated through blood pressure, we performed a conditional analysis. We conditioned the IA summary statistics on summary statistics for blood pressure, similar to using blood pressure as covariate in the IA genome-wide association study. Conditioning was done using multi-trait conditional and joint (mtCOJO) analysis[53] using its default setting using summary statistics for systolic and diastolic blood pressure measurements from the UK Biobank together (http://www.nealelab.is/uk-biobank/). European-ancestry genotypes from the 1000 Genomes project were used as reference panel.

### *Benchmarking the role of CNNM2 in blood pressure and IA*

Since we limited our primary analyses to anti-epileptic drug targets and thus other genes with a stronger effect on IA that we did not include may be present, we benchmarked the importance of *CNNM2* in IA and blood pressure to all other genes. We conducted MR with expression of each gene

in tibial artery, and in whole blood as exposures, and blood pressure, and IA as outcomes. To allow a fair comparison of effect sizes, we standardized genome-wide association effect size of gene expression to mean=0, variance=1 for each gene separately. Then, we performed an inverse variance weighted MR analysis of expression of each gene separately on IA, and on systolic and diastolic blood pressure in both tibial artery and blood.

## Supplementary Figures and Figure legends

**Supplementary Fig. S1. Overlap of genetic association signals between gene expression and intracranial aneurysm risk.** Plots are shown for A) *CNNM2* expression in tibial artery, and intracranial aneurysms (IA), B) *CNNM2* expression in blood, and IA, C) *IRF1* expression in blood, and IA. Gene expression traits are shown in red, IA in black. X-axis depicts chromosomal position (GRCh37), Y-axis indicates -$\log_{10}$(P-value).

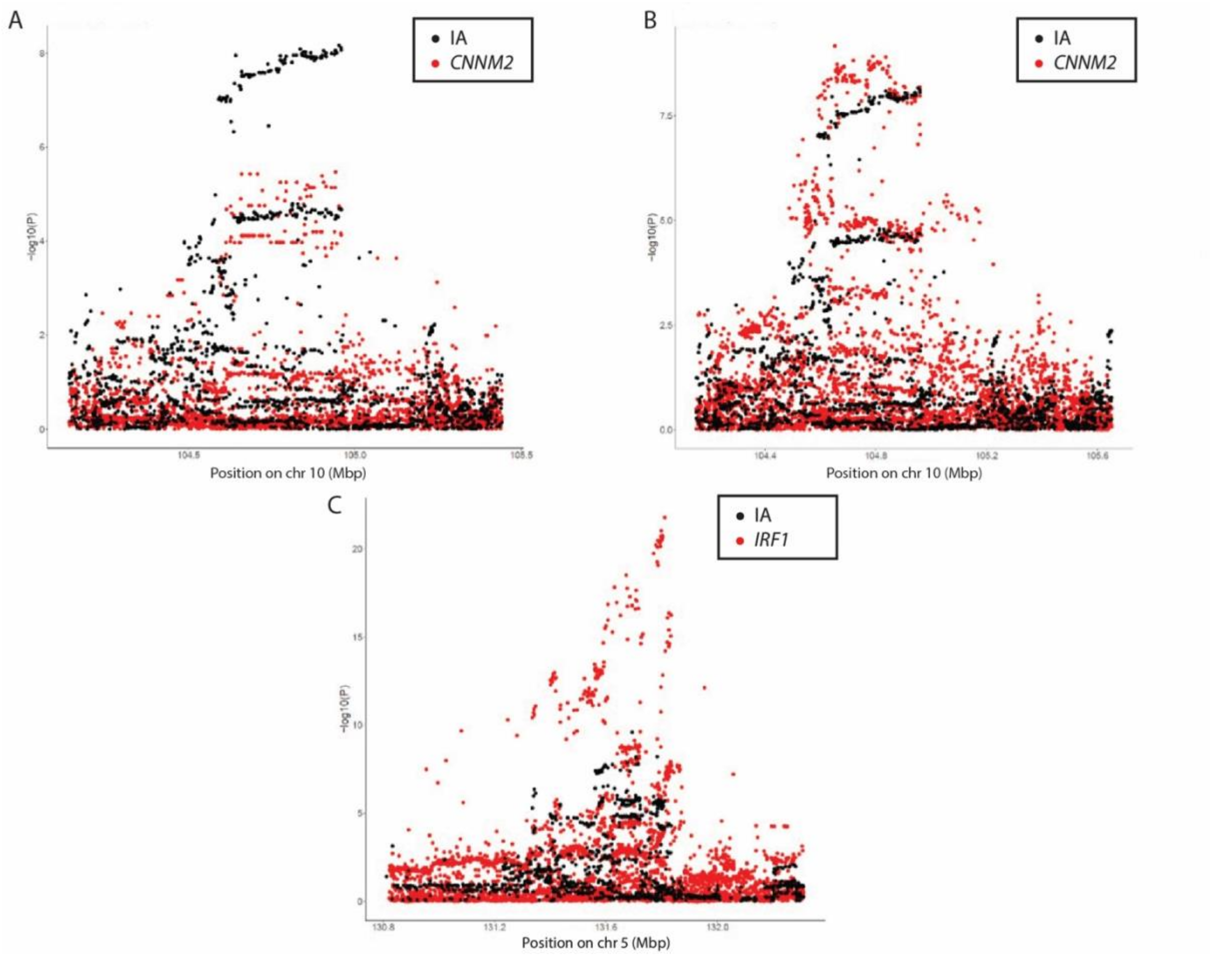

**Supplementary Fig. S2. Gene-wise comparison of Mendelian randomization (MR) effects on blood pressure and on intracranial aneurysms in tibial artery.** Each point represents a gene of which genetically proxied expression has an MR effect on A) diastolic blood pressure or B) systolic blood pressure with MR P-value < 0.001. Error bars and shaded area: 95% confidence intervals. *CNNM2* is highlighted. Lines are based on a linear regression of the per-gene MR effects of IA on blood pressure including all genes (red) and excluding *CNNM2*. Figure on next page.

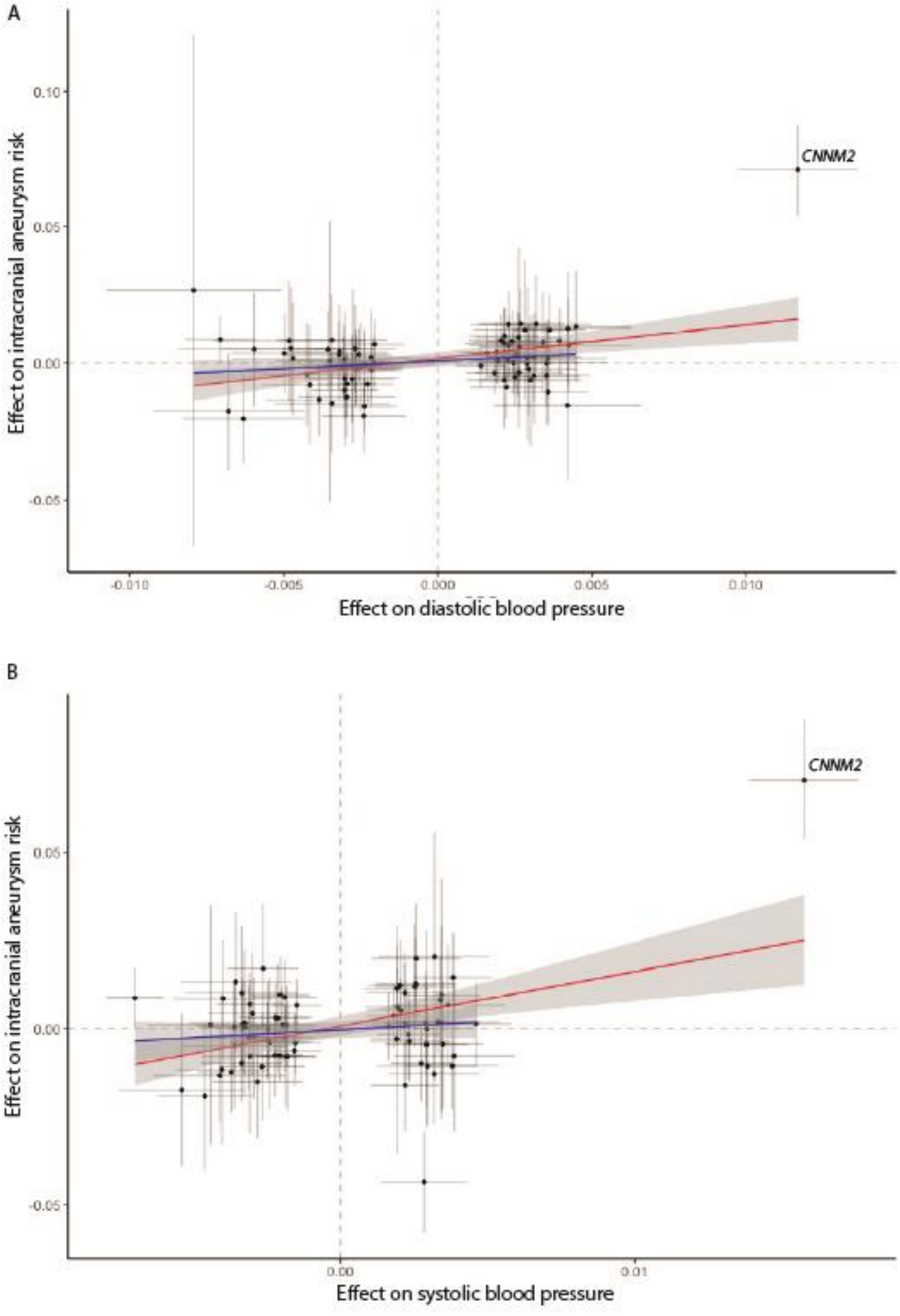
A
CNNM2
Effect on intracranial aneurysm risk
0.10
0.05
0.00
-0.05
-0.010
-0.005
0.000
0.005
0.010
Effect on diastolic blood pressure
B
CNNM2
Effect on intracranial aneurysm risk
0.05
0.00
-0.05
0.00
0.01
Effect on systolic blood pressure

**Supplementary Fig. S3. Gene-wise comparison of Mendelian randomization (MR) effects on blood pressure and on intracranial aneurysms in blood.** Each point represents a gene of which genetically proxied expression has an MR effect on A) diastolic blood pressure or B) systolic blood pressure with P-value < 0.001. Error bars and shaded area: 95% confidence intervals. *CNNM2* is highlighted. Lines are based on a linear regression of the per-gene MR effects of IA on blood pressure including all genes (red) and excluding *CNNM2*. Figure on next page.

## Checklist

| Item No | Section | Checklist item | Relevant text from manuscript |
|---|---|---|---|
| 1 | **Title and abstract** | Indicate mendelian randomisation (MR) as the study's design in the title and/or the abstract if that is a main purpose of the study | Title, and abstract text: We first assessed if genetic predisposition for epilepsy may be causally related to IA using two-sample Mendelian randomization (MR). Next, using two-sample MR and colocalization analyses we assessed if IA-associated genes encode for anti-epileptic drug targets. |
| | **Introduction** | | |
| 2 | Background | Explain the scientific background and rationale for the reported study. What is the exposure? Is a potential causal association between exposure and outcome plausible? Justify why MR is a helpful method to address the study question | In the largest GWAS to date, including 10,754 cases and 306,882 controls, drug target enrichment showed pleiotropy between IA and anti-epileptic drugs.[9] We cannot yet explain what drives this pleiotropy. Understanding the mechanisms involved in this pleiotropy will lead to further understanding of the disease-causing mechanisms of IA and may help to identify |

| Item No | Section | Checklist item | Relevant text from manuscript |
|---|---|---|---|
| | | | methods of IA prevention using anti-epileptics or related drugs.<br><br>Therefore, the aim of our study was to gain insight in the previously established pleiotropy between IA and anti-epileptic drugs. We hypothesized that there are two potential explanations for the observed pleiotropy: there may be a causal effect of genetic predisposition to epilepsy on IA and/or there may be a causal effect of expression levels of genes encoding effective anti-epileptic drugs on IA risk. These hypotheses can both be tested using two-sample mendelian randomization (MR) and colocalization analyses. |
| 3 | Objectives | State specific objectives clearly, including prespecified causal hypotheses (if any). State that MR is a method that, under specific assumptions, | Therefore, the aim of our study was to gain insight in the previously established pleiotropy between IA and anti-epileptic drugs. |

| Item No | Section | Checklist item | Relevant text from manuscript |
|---|---|---|---|
| | | intends to estimate causal effects | |
| | **Methods** | | |
| 4 | Study design and data sources | Present key elements of the study design early in the article. Consider including a table listing sources of data for all phases of the study. For each data source contributing to the analysis, describe the following: | Paragraph ‘Study design |
| | a) | Setting: Describe the study design and the underlying population, if possible. Describe the setting, locations, and relevant dates, including periods of recruitment, exposure, follow-up, and data collection, when available. | Original studies, reported in paragraph ‘Dataset description’ |

| Item No | Section | Checklist item | Relevant text from manuscript |
|---|---|---|---|
| | b) | Participants: Give the eligibility criteria, and the sources and methods of selection of participants. Report the sample size, and whether any power or sample size calculations were carried out prior to the main analysis | Paragraph ‘Dataset description’ |
| | c) | Describe measurement, quality control, and selection of genetic variants | Instruments (i.e., uncorrelated variants) for MR should be associated with the exposure. To achieve this, a threshold for association p-value is typically applied. The downside of a stringent threshold is that it limits the number of variants available thereby potentially decreasing accuracy. This means that no single optimal p-value threshold exists. Therefore, we used two p-value thresholds for the association of a variant with the exposure: $p < 5\times10^{-5}$ and $p < 5\times10^{-8}$. After this selection the genetic variants were clumped to select LD-independent |

| Item No | Section | Checklist item | Relevant text from manuscript |
|---|---|---|---|
| | | | instruments (LD $r^2 > 0.01$, or outside 10 megabase window to include) using the 1000 Genomes European reference panel. The analysis method was performed using the R packages TwoSampleMR (version 0.5.5) and MRInstruments (version 0.3.2). |
| | d) | For each exposure, outcome, and other relevant variables, describe methods of assessment and diagnostic criteria for diseases | Original studies, reported in paragraph 'Dataset description' |
| | e) | Provide details of ethics committee approval and participant informed consent, if relevant | This study was based on aggregate data and therefore no institutional review board approval was necessary. All prior studies providing aggregate data received ethical approval and obtained informed consent from all participants. |

| Item No | Section | Checklist item | Relevant text from manuscript |
|---|---|---|---|
| 5 | Assumptions | Explicitly state the three core instrumental variable assumptions for the main analysis (relevance, independence, and exclusion restriction), as well assumptions for any additional or sensitivity analysis | In other to infer causality in MR, a genetic variant causing the outcome through the exposure (and therefore being a valid instrumental variable) must meet core three assumptions: 1) genetic variants have a causal effect on the outcome, 2) the genetic variants affect the outcome only through the exposure, and 3) the genetic variants are not associated with a confounding factor influencing both the exposure and outcome. |
| 6 | Statistical methods: main analysis | Describe statistical methods and statistics used | |
| | a) | Describe how quantitative variables were handled in the analyses (that is, scale, units, model) | Original studies, reported in paragraph 'Dataset description' |
| | b) | Describe how genetic variants were handled in the analyses and, if applicable, | Original studies, reported in paragraph 'Dataset description' |

| Item No | Section | Checklist item | Relevant text from manuscript |
|---|---|---|---|
| | | how their weights were selected | |
| | c) | Describe the MR estimator (eg, two stage least squares, Wald ratio) and related statistics. Detail the included covariates and, in the case of two sample MR, whether the same covariate set was used for adjustment in the two samples | The inverse variance weighted (IVW) MR method was selected as our main MR analysis method. Two linkage disequilibrium (LD)-independent variants are needed to perform IVW. IVW estimates are accurate if all genetic variants included conform to the MR assumptions of no heterogeneity. If only a single LD-independent variant was available, we used a Wald ratio test to quantify its MR effect but did not use it for further analysis since with a single genetic variant no test for pleiotropy can be performed, making it impossible to assess reliability of the estimates. MR-PRESSO was used in addition to exclude eQTL and pQTL where pleiotropic outliers influenced the IVW estimate. The MR-PRESSO method is an extension of the IVW method and is robust for horizontal pleiotropy amongst the genetic variants compared to IVW, by |

| Item No | Section | Checklist item | Relevant text from manuscript |
|---|---|---|---|
| | | | identifying and removing pleiotropic outliers from the analysis.[28, 29] First, MR-PRESSO was used to identify pleiotropic outliers, which were then removed, and MR-PRESSO was subsequently used to calculate the MR effect using R package MRPRESSO (version 1.0). Empirical p-value was obtained by 1000 permutations. At least four LD-independent variants are needed to perform MR-PRESSO. |
| | d) | Explain how missing data were addressed | n/a |
| | e) | If applicable, indicate how multiple testing was addressed | n/a |
| 7 | Assessment of assumptions | Describe any methods or prior knowledge used to assess the assumptions or justify their validity | Instruments (i.e., uncorrelated variants) for MR should be associated with the exposure. To achieve this, a threshold for association p-value is typically applied. The downside of a stringent threshold is that it limits the |

| Item No | Section | Checklist item | Relevant text from manuscript |
| --- | --- | --- | --- |
| | | | number of variants available thereby potentially decreasing accuracy. This means that no single optimal p-value threshold exists. Therefore, we used two p-value thresholds for the association of a variant with the exposure: $p < 5\times10^{-5}$ and $p < 5\times10^{-8}$. After this selection the genetic variants were clumped to select LD-independent instruments (LD $r^2 > 0.01$, or outside 10 megabase window to include) using the 1000 Genomes European reference panel. The analysis method was performed using the R packages TwoSampleMR (version 0.5.5) and MRInstruments (version 0.3.2).[30]<br><br>Drug targets with a p-value < 0.05 for the IVW method, that did not have a p-value > 0.05 for MR-PRESSO (since this analysis requires two additional independent variants which were not available for all QTL, absence of an MR-PRESSO estimate was no disqualifying threshold) were selected for colocalization analysis. |

| Item No | Section | Checklist item | Relevant text from manuscript |
|---|---|---|---|
| | | | *Sensitivity analyses*<br>Several sensitivity analyses were performed to determine the presence of heterogeneity and pleiotropy for genetic variants or studies that were included in the analysis. We performed the following analyses: MR-Egger, weighted median, weighted mode, simple mode, Cochran's Q test, and the Steiger directionality test. More details and rationale can be found in the Supplementary Methods. |
| 8 | Sensitivity analyses and additional analyses | Describe any sensitivity analyses or additional analyses performed (eg, comparison of effect estimates from different approaches, independent replication, bias analytic techniques, validation of instruments, simulations) | Paragraph 'Sensitivity analyses' |

| Item No | Section | Checklist item | Relevant text from manuscript |
|---|---|---|---|
| 9 | Software and pre-registration | | |
| | a) | Name statistical software and package(s), including version and settings used | The analysis was performed using the R packages TwoSampleMR (version 0.5.5) and MRInstruments (version 0.3.2).<br><br>we performed colocalization analysis using the R package Coloc (version 3.2-1). |
| | b) | State whether the study protocol and details were pre-registered (as well as when and where) | n/a |
| | **Results** | | |
| 10 | Descriptive data | | |
| | a) | Report the numbers of individuals at each stage of included studies and reasons for exclusion. | Paragraph ‘Dataset description’ |

| Item No | Section | Checklist item | Relevant text from manuscript |
|---|---|---|---|
| | | Consider use of a flow diagram | |
| | b) | Report summary statistics for phenotypic exposure(s), outcome(s), and other relevant variables (eg, means, SDs, proportions) | Original studies, reported in paragraph 'Dataset description' |
| | c) | If the data sources include meta-analyses of previous studies, provide the assessments of heterogeneity across these studies | n/a |
| | d) | For two sample MR:<br>i. Provide justification of the similarity of the genetic variant-exposure associations between the exposure and outcome samples<br>ii. Provide information on | These was no known sample overlap between studied jointly analyzed with MR. |

| Item No | Section | Checklist item | Relevant text from manuscript |
| --- | --- | --- | --- |
| | | the number of individuals who overlap between the exposure and outcome studies | |
| 11 | Main results | | |
| | a) | Report the associations between genetic variant and exposure, and between genetic variant and outcome, preferably on an interpretable scale | Table 2 |
| | b) | Report MR estimates of the association between exposure and outcome, and the measures of uncertainty from the MR analysis, on an interpretable scale, such as odds ratio or relative risk per SD difference | Results page 9 and 10 describe the main findings in detail. The MR estimates of our top findings are shown in Table 1, and Supplementary Tables S2-S5 |

| Item No | Section | Checklist item | Relevant text from manuscript |
|---|---|---|---|
| | c) | If relevant, consider translating estimates of relative risk into absolute risk for a meaningful time period | n/a |
| | d) | Consider plots to visualise results (eg, forest plot, scatterplot of associations between genetic variants and outcome *v* between genetic variants and exposure) | In Figure 1 the MR effects and uncertainty of our main findings are plotted. |
| 12 | Assessment of assumptions | | |
| | a) | Report the assessment of the validity of the assumptions | Results of sensitivity analyses are shown in Supplementary Tables S2-S5. The Cochran's Q test indicated there was no evidence for heterogeneity in the MR analyses of *CNNM2* and *IRF1* expression levels on IA risk (Supplementary Table S6). The Steiger directionality confirmed that the genetic |

| Item No | Section | Checklist item | Relevant text from manuscript |
| --- | --- | --- | --- |
| | | | associations were consistent with a causal effect of *IRF1* and *CNNM2* levels on IA risk, and not in the opposite direction (Supplementary Table S7). |
| | b) | Report any additional statistics (eg, assessments of heterogeneity across genetic variants, such as $I^2$, Q statistic, or E value) | Supplementary Table S6 |
| 13 | Sensitivity analyses and additional analyses | | |
| | a) | Report any sensitivity analyses to assess the robustness of the main | Supplementary Tables S2-S5 |

| Item No | Section | Checklist item | Relevant text from manuscript |
|---|---|---|---|
| | | results to violations of the assumptions | |
| | b) | Report results from other sensitivity analyses or additional analyses | Supplementary Tables S6 |
| | c) | Report any assessment of direction of causal association (eg, bidirectional MR) | Supplementary Table S7 |
| | d) | When relevant, report and compare with estimates from non-MR analyses | n/a |
| | e) | Consider additional plots to visualise results (eg, leave-one-out analyses) | Supplementary Figure S1 shows scatterplots of regional association statistics of intracranial aneurysms, *CNNM2* expression, and *IRF1* expression, to support our colocalization analyses. |
| | **Discussion** | | |

| Item No | Section | Checklist item | Relevant text from manuscript |
|---|---|---|---|
| 14 | Key results | Summarise key results with reference to study objectives | From: Discussion paragraph 1 (page 10):<br><br>We found that the previously established pleiotropy between genetic risk for IA, and the genes targeted by anti-epileptic drugs, is not driven directly by an increased predisposition for epilepsy also affecting IA risk but is partly explained by variation in gene expression of *CNNM2*. Higher gene expression levels of *CNNM2,* a gene of which knockdown mimics gene expression patterns after anti-epileptic drug administration, in arterial tissue is consistent with a causal increase in the risk of IA. |
| 15 | Limitations | Discuss limitations of the study, taking into account the validity of the instrumental variable assumptions, other sources of potential bias, and imprecision. Discuss both direction and magnitude of | From: Discussion paragraph 6 (page 11-12):<br><br>This study has certain limitations. First, not all anti-epileptic drugs were present in the CMap database or DGIbd, and the drug valpromide was not listed in either database. Since many drugs had overlapping targets, we expect that most anti-epileptic drug targets were included. Second, |

| Item No | Section | Checklist item | Relevant text from manuscript |
| --- | --- | --- | --- |
| | | any potential bias and any efforts to address them | expression of most genes is influenced by local genetic interaction (cis-eQTL).[14] This means that most variants influencing gene expression are in proximity and thus often in LD. Therefore, only a small number of LD-independent variants could be used for MR, leading to relatively few genes with sufficient LD-independent variants for MR and a relatively large imprecision in MR estimates for the remaining genes. Compared to eQTL, variants affecting protein levels (pQTL) are more often distant from the encoding gene.[42] This resulted in a greater number of SNPs to be included in our pQTL analyses, partially overcoming the limitation of few instruments. Finally, findings by MR infer a potential causal effect of genetic predisposition of an exposure on an outcome but does not provide evidence that changes in exposure by therapeutical intervention will substantially influence the outcome. Therefore, additional research is |

| Item No | Section | Checklist item | Relevant text from manuscript |
| --- | --- | --- | --- |
| | | | needed to determine the effect of the usage of anti-epileptic drugs on IA risk. |
| 16 | Interpretation | | |
| | a) | Meaning: Give a cautious overall interpretation of results in the context of their limitations and in comparison with other studies | From: Discussion paragraph 6 (page 11-12):<br><br>Finally, findings by MR infer a potential causal effect of genetic predisposition of an exposure on an outcome but does not provide evidence that changes in exposure by therapeutical intervention will substantially influence the outcome. Therefore, additional research is needed to determine the effect of the usage of anti-epileptic drugs on IA risk |
| | b) | Mechanism: Discuss underlying biological mechanisms that could drive a potential causal association between the investigated exposure and the outcome, and whether | Discussion paragraphs 2, 3, and 4 |

| Item No | Section | Checklist item | Relevant text from manuscript |
|---|---|---|---|
| | | the gene-environment equivalence assumption is reasonable. Use causal language carefully, clarifying that instrumental variable estimates may provide causal effects only under certain assumptions | |
| | c) | Clinical relevance: Discuss whether the results have clinical or public policy relevance, and to what extent they inform effect sizes of possible interventions | From: Discussion paragraph 7 (page 12): We found a causal effect of increased gene expression of anti-epileptic drug target CNNM2 on IA. The effect could not be explained by increased blood pressure. Anti-epileptic drugs phenytoin, valproic acid and *carbamazepine* are expected to lower CNNM2 levels which subsequently may lead to a lower IA risk. Follow-up studies are required to investigate whether persons exposed to these anti-epileptic drugs have indeed a lower risk of unruptured IA and/or aneurysmal subarachnoid haemorrhage and |

| Item No | Section | Checklist item | Relevant text from manuscript |
|---|---|---|---|
| | | | how variation in *CNNM2* expression can lead to IA. (…) This study highlights *CNNM2* as a relevant drug target for IA. |
| 17 | Generalisability | Discuss the generalisability of the study results (a) to other populations, (b) across other exposure periods/timings, and (c) across other levels of exposure | Finally, findings by MR infer a potential causal effect of genetic predisposition of an exposure on an outcome but does not provide evidence that changes in exposure by therapeutical intervention will substantially influence the outcome. Therefore, additional research is needed to determine the effect of the usage of anti-epileptic drugs on IA risk. |
| | **Other information** | | |
| 18 | Funding | Describe sources of funding and the role of funders in the present study and, if applicable, sources of funding for the databases and original study or studies | Paragraph 'Sources of funding' |

| Item No | Section | Checklist item | Relevant text from manuscript |
|---|---|---|---|
| | | on which the present study is based | |
| 19 | Data and data sharing | Provide the data used to perform all analyses or report where and how the data can be accessed, and reference these sources in the article. Provide the statistical code needed to reproduce the results in the article, or report whether the code is publicly accessible and if so, where | All data is provided in the Tables and Supplementary Tables. Intermediate files can be requested from the authors. |
| 20 | Conflicts of interest | All authors should declare all potential conflicts of interest | Paragraph ‘Disclosures’ |
